# Exploring *Tectona grandis* Linn. f. Leaf Extract as a Functional Feed Additive with Antioxidant and Nutraceutical Potential for Livestock

**DOI:** 10.3390/ani15233498

**Published:** 2025-12-04

**Authors:** Nattaya Montri, Metha Wanapat, Sungchhang Kang, Seangla Cheas, Anusorn Cherdthong, Pongsatorn Gunun, Nirawan Gunun, Suban Foiklang, Phongthorn Kongmun, Dutsadee Srithat, Pongsathorn Tongkasee, Sineenart Polyorach

**Affiliations:** 1 Office of Administrative Interdisciplinary Program on Agricultural Technology, School of Agricultural Technology, King Mongkut’s Institute of Technology Ladkrabang, Bangkok 10520, Thailand; nattaya.mo@kmitl.ac.th; 2 Tropical Feed Resources Research and Development Center (TROFREC), Department of Animal Science, Faculty of Agriculture, Khon Kaen University, Khon Kaen 40002, Thailand; metha@kku.ac.th (M.W.); seangla.c@kkumail.com (S.C.); anusornc@kku.ac.th (A.C.); 3 Agricultural Unit, Department of Education, National Institute of Education, Phnom Penh 12207, Cambodia; ksungchhang@yahoo.com; 4 Department of Animal Science, Faculty of Natural Resources, Rajamangala University of Technology Isan, Sakon Nakhon Campus, Phangkhon, Sakon Nakhon 47160, Thailand; pongsatorn.gu@rmuti.ac.th; 5 Department of Animal Science, Faculty of Technology, Udon Thani Rajabhat University, Udon Thani 41000, Thailand; nirawan.gu@udru.ac.th; 6 Faculty of Animal Science and Technology, Maejo University, Chiang Mai 50290, Thailand; bungung@hotmail.com; 7 Department of Animal Science, Faculty of Agriculture, Kasetsart University, Bangkok 10900, Thailand; fagrptk@ku.ac.th; 8 Thai Traditional Medicine, Faculty of Natural Resources, Rajamangala University of Technology Isan, Sakon Nakhon Campus, Sakon Nakhon 47160, Thailand; dutsadee.si@rmuti.ac.th; 9 Department of Animal Production Technology and Fisheries, Faculty of Agricultural Technology, King Mongkut’s Institute of Technology Ladkrabang, Bangkok 10520, Thailand

**Keywords:** *Tectona grandis*, phytochemical profile, antioxidant activity, functional feed, livestock nutrition

## Abstract

This study investigated the phytochemical composition, antioxidant activity, and safety of *Tectona grandis* leaf extracts at four maturity stages. Ethanolic extracts were screened for secondary metabolites and analyzed using GC–MS, together with heavy metal determination, phenolic profiling, and antioxidant assays. Preliminary phytochemical screening revealed the presence of alkaloids, flavonoids, glycosides, tannins, saponins, and coumarins, with clear variation among leaf stages. GC–MS analysis detected multiple bioactive constituents, including phytol, n-decanoic acid, and heptadecenal. These compounds have been previously reported to exhibit antioxidant, antimicrobial, and anti-inflammatory properties. Heavy metal analysis showed very low cadmium concentrations (0.001–0.004 mg/kg), undetectable lead levels (<LOD), and trace levels of arsenic concentrations (0.012–0.018 mg/kg), all of which were within safe limits for plant materials. Quantitative assays demonstrated that mature leaves contained the highest total phenolic (8.751 ± 0.018 mg GAE/g DW), total flavonoid (0.359 ± 0.017 mg QE/g DW), and condensed tannin (0.303 ± 0.000 mg CE/g DW) contents. Correspondingly, mature-leaf extracts exhibited the greatest antioxidant capacity, showing 95.88 ± 0.12% inhibition of oxidant activity. The ethanolic extracts exhibited IC_50_ values ranging from 22.93–50.35 mg/mL, whereas the ascorbic acid standard showed an IC_50_ of 0.0767 mg/mL, confirming the expected lower potency of crude plant extracts. The strong correlation between phenolic accumulation and antioxidant activity suggests enhanced defense against oxidative stress. The absence of detectable heavy metal contamination, together with the high polyphenolic content, suggests that *T. grandis* leaves may serve as a safe natural source of antioxidants. These findings demonstrate the strong antioxidant potential of *T. grandis* leaf extracts, providing a basis for future studies evaluating their functional applications in biological systems.

## Simple Summary

Teak (*Tectona grandis* Linn. f.) is widely known as a tropical hardwood tree, but its leaves also contain valuable bioactive compounds with strong antioxidant properties. This study explored the phytochemical composition and antioxidant activity of *T. grandis* leaf extracts to assess their potential as natural feed additives for livestock. Mature leaves showed the highest levels of phenolic, flavonoid, and tannin compounds. They also exhibited the strongest antioxidant capacity and the lowest IC_50_ values. No toxic heavy metals were detected, indicating that the extracts were chemically safe within the scope of this analysis. Overall, the results provide baseline evidence that *T. grandis* leaves are a natural source of antioxidant compounds and warrant further investigation for possible applications in animal nutrition and other biological systems.

## 1. Introduction

*Tectona grandis* Linn. f. (family Verbenaceae), commonly known as teak, is a highly valued tropical hardwood. It is widely distributed across South and Southeast Asia, including India, Laos, Myanmar, northern Thailand, and parts of China [1]. In addition to its value as a timber species, teak has long been used in Ayurvedic and traditional herbal medicine. Different plant parts have been used to relieve bronchitis, headache, diabetes, and inflammation. Teak plant parts also exhibit diuretic and laxative properties [2,3]. These observations suggest that the species contains diverse bioactive compounds with potential nutritional relevance.

Several in vitro studies have shown that teak leaves contain secondary metabolites with antioxidant, antibacterial, antifungal, anti-inflammatory, and anti-diabetic activities. Han et al. [2] identified 26 compounds in methanolic extracts with strong radical-scavenging capacity. Lankaa and Parimala [4] reported antimicrobial activity in ethanolic and aqueous extracts. Kamath and Shabaraya [5] observed inhibition of *Staphylococcus aureus*, *Escherichia coli*, and *Pseudomonas aeruginosa*. Most previous studies used only one solvent or investigated a limited group of compounds. Comparisons across leaf maturity stages are scarce, even though leaf age strongly influences metabolic activity, structural development, and secondary-metabolite biosynthesis. Young leaves typically synthesize more defensive compounds. In contrast, mature leaves accumulate higher concentrations of phenolics, flavonoids, and tannins [2,5].

For livestock nutrition, the phytochemical composition of teak leaves is important because of their functional properties. Natural antioxidants help counter oxidative stress, which can affect immunity, growth, reproduction, and metabolic efficiency in animals [6]. Oxidative stress occurs when pro-oxidant production exceeds antioxidant defenses. Diets enriched with phenolics and flavonoids enhance antioxidant capacity, reduce lipid peroxidation, and modulate inflammatory pathways [7,8]. At moderate levels, tannins may promote protein-sparing effects in ruminants by increasing rumen bypass of dietary protein [2,9]. Plants with high levels of these compounds have potential as natural alternatives to synthetic antioxidants in feed.

Despite this potential, the influence of leaf maturity on the functional value of *T. grandis* leaves remains unclear. Published studies have not described how phytochemical concentrations change across developmental stages. Information on heavy metal residues is also limited. Such data are essential when evaluating potential pharmacological or feed applications. A better understanding of how leaf maturity affects chemical composition and antioxidant activity is necessary to determine the stage with the greatest functional value.

The present study was designed to address these gaps. Five objectives were established: (i) screening preliminary phytochemical constituents of ethanolic extracts, (ii) identifying bioactive compounds using GC–MS, (iii) quantifying phenolic, flavonoid, and tannin concentrations, (iv) assessing heavy metal residues, and (v) evaluating antioxidant activity across four leaf-maturity stages. It was hypothesized that mature leaves would contain higher levels of key phytochemicals and exhibit stronger antioxidant activity than younger leaves. This information is important for identifying the maturity stage with the highest potential for pharmacological applications or use as a natural antioxidant source in animal nutrition.

## 2. Materials and Methods

### 2.1. Plant Sample Collection and Authentication

Leaves of *Tectona grandis* Linn. f. were collected from the Plant Genetic Conservation Project under the Royal Initiative of Her Royal Highness Princess Maha Chakri Sirindhorn in Tha Sae District, Chumphon Province, Thailand (10°53′33.3″ N, 99°15′10.2″ E). Leaves were handpicked from pruned branches at the first- to ninth-leaf positions.

Four maturity stages were defined using leaf position and visible characteristics. Leaves were counted from the shoot tip as positions 1 to 9. Young leaves were collected from positions 1–3 and were small, soft, and light green. Medium leaves were taken from positions 4–6 and were larger, firmer, and uniformly green. Mature leaves were taken from positions 7–9 and were fully expanded, thick, and dark green. The mixed category contained an equal number of leaves from positions 1–9 on the same branch.

A voucher specimen was authenticated and deposited at the Bangkok Herbarium (BK), following procedures described by Ogunmefun et al. [10].

### 2.2. Plant Extracts Preparation

The extraction method followed Longbap et al. [11] with modifications. Leaves from each maturity stage were washed with distilled water and air-dried at room temperature. They were then oven-dried at 50 °C for 24 h, ground into powder, and passed through a 60-mesh sieve. The powder was dried again at 45 °C for 48 h until constant weight to remove residual moisture.

Each powdered sample was extracted by maceration in ethanol (95%; Merck KGaA, Darmstadt, Germany) at a 1:5 (*w*/*v*) ratio (1 g leaf powder per 5 mL solvent). Samples were placed in sealed amber bottles and kept at room temperature (28–30 °C) for seven days. Bottles were gently shaken twice daily (about 1 min per time) to improve solvent–solid contact. The mixture was then filtered through Whatman No. 1 filter paper (Cytiva, Marlborough, MA, USA) and the filtrate was concentrated under reduced pressure using a rotary evaporator at 60 °C and 110 rpm. The semisolid extracts were stored in airtight amber containers at 15 °C until analysis.

### 2.3. Preliminary Phytochemical Screening

Qualitative screening of the ethanolic extracts was completed using standard colorimetric and precipitation tests based on Longbap et al. [11]. The procedures enabled the detection of major secondary metabolite groups, including alkaloids, flavonoids, tannins, saponins, terpenoids, and phenolic compounds.

All analyses were performed using three biological replicates, and each assay was measured in triplicate to ensure precision and consistency.

### 2.4. Gas Chromatography-Mass Spectroscopy (GC-MS)

Phytochemical profiling of the ethanolic extracts was carried out using gas chromatography–mass spectrometry GC–MS system (GC-MS-QP2020 Ultra, Shimadzu Corporation, Kyoto, Japan) following the modified procedure of Alabi and Oyeku [12]. Dried extracts were re-dissolved in analytical-grade ethanol (1 mg/mL), vortexed, and filtered through a 0.22 µm PTFE syringe filter prior to analysis. All samples were analyzed using a GC–MS system (GC-MS-QP2020 Ultra; Shimadzu Corporation, Kyoto, Japan) equipped with an SH-Rtx-5MS capillary column (30 m × 0.32 mm i.d., 0.25 µm film thickness; Restek Corporation, Bellefonte, PA, USA). Helium served as the carrier gas at a constant flow rate of 1.37 mL/min. The injector operated in split mode (50:1) at 250 °C. The oven program began at 60 °C (2 min hold), increased to 180 °C at 10 °C/min, then to 280 °C at 5 °C/min, with a final hold of 10 min. The interface temperature was maintained at 280 °C. Mass spectra were recorded under electron-impact ionization at 70 eV with a scan range of m/z 40–550 and a solvent delay of 3 min.

Compound identification was based on comparison with the NIST17 spectral library, using a minimum similarity threshold of 80%. Retention indices were also calculated and compared with published values to strengthen identification confidence. Only compounds meeting these criteria were included. Each extract was analyzed in triplicate using independent biological samples.

To ensure analytical reliability, solvent blanks and system blanks (ethanol-only injections) were run under the same GC–MS conditions as the samples. Peaks detected in blank injections, or known to originate from column bleed, solvent impurities, or environmental background, were flagged as potential contaminants. These peaks were excluded from biological interpretation. Only peaks absent in blanks and fulfilling the identification criteria (similarity ≥80% and RI match) were considered authentic constituents of *T. grandis* extracts.

### 2.5. Determination of Metal Residues

Heavy metal residues in *T. grandis* leaf extracts were determined using atomic absorption spectroscopy (AAS, Shimadzu AA-7000, Shimadzu Corporation, Kyoto, Japan). Flame AAS (FAAS) was used for cadmium (Cd, 228.8 nm) and lead (Pb, 217.0 nm), while graphite-furnace AAS (GFAAS) was applied for arsenic (As, 193.7 nm) following the procedures described by Lim et al. [13]. Sample digestion followed the AOAC acid-digestion method [14].

One gram of dried sample was placed in a 50 mL round-bottom flask with 12 mL concentrated HNO_3_ (65%) and 4 mL HCl (37%) (Merck KGaA, Darmstadt, Germany). The mixture was heated gently until a clear solution was obtained. It was then cooled to room temperature and diluted to 100 mL with deionized water. Calibration standards were prepared in an acidic matrix consisting of 12% (*v*/*v*) HNO_3_, 4% (*v*/*v*) HCl, and 84% (*v*/*v*) deionized water. Standard concentration ranges were 10–25 ppb for As, 0.05–0.6 ppm for Cd, and 0.5–20 ppm for Pb.

Instrumental baseline correction was applied automatically by the atomic absorption spectrophotometer (AAS system). Negative values generated after baseline adjustment were interpreted as concentrations below the method detection limit (LOD). The LOD and LOQ for each metal were calculated using ten procedural blanks. These were based on 3× and 10× the standard deviation of blank signals, respectively. Values falling below the LOD were reported as not detected (ND) [15].

All metal analyses were performed using three biological replicates, with each measurement conducted in triplicate to ensure analytical precision.

### 2.6. Determination of Bioactive Constituents

The total phenolic, flavonoid, condensed tannin, and total tannin contents of *Tectona grandis* ethanolic extracts were analyzed using standard spectrophotometric techniques. All measurements were performed using a UV–Vis spectrophotometer (SPECORD 200; Analytik Jena GmbH, Jena, Germany). The total phenolic content (TPC) was determined using the Folin–Ciocalteu method [16]. A 0.5 mL aliquot of extract was mixed with 2.5 mL of diluted Folin–Ciocalteu reagent (1:10 *v*/*v*) and 4 mL of sodium carbonate (7.5% *w*/*v*). The mixture was incubated at 45 °C for 30 min, after which absorbance was measured at 760 nm. Gallic acid (2.5–250 µg mL^−1^) served as the standard, and results were expressed as milligrams of gallic acid equivalents (mg GAE) per gram of extract.

The total flavonoid content (TFC) was quantified using the aluminum chloride colorimetric method [17]. The absorbance of each reaction mixture was read at 510 nm and compared against a quercetin standard curve, with results expressed as milligrams of quercetin equivalents (mg QE) per gram of extract. Condensed tannin content (CTC) was measured by the vanillin–HCl assay following the procedure of Broadhurst and Jones [18,19]. A mixture consisting of 3 mL of vanillin solution (4% in methanol), 0.4 mL of the extract, and 1.5 mL of concentrated HCl was incubated for 15 min, after which the absorbance was recorded at 500 nm. The CTC values were expressed as milligrams of catechin equivalents (mg CE) per gram of extract.

The total tannin content (TTC) was also determined using the Folin–Ciocalteu method [20]. A 0.1 mL aliquot of extract was diluted with 7.5 mL of distilled water, followed by the addition of 0.5 mL of Folin reagent and 1 mL of sodium carbonate (35% *w*/*v*). The total volume was adjusted to 10 mL with distilled water and incubated for 30 min at room temperature. Absorbance was measured at 750 nm, and the TTC values were expressed as milligrams of gallic acid equivalents (mg GAE) per gram of extract.

These quantifications provided an overview of the phenolic and tannin compounds that contribute to the antioxidant potential of *T. grandis* leaves, which are of particular interest for their possible application as natural antioxidant additives in animal feed.

### 2.7. Antioxidant Screening

#### 2.7.1. DPPH Radical-Scavenging Assay

The antioxidant activity of the ethanolic extracts was assessed using the 2,2-diphenyl-1-picrylhydrazyl (DPPH•) method following Siddartha et al. [21] and Eugenio et al. [22], with modifications to ensure reproducibility. The DPPH• reagent was obtained from Sigma–Aldrich (St. Louis, MO, USA), and absolute ethanol was purchased from Merck KGaA (Darmstadt, Germany). A stock solution of DPPH• (0.5 mM) was prepared fresh in absolute ethanol and protected from light. Extract solutions were prepared at different concentrations and filtered through a 0.22 µm membrane before use. All solutions were prepared fresh on the day of analysis.

For each reaction, 0.5 mL of the extract solution was mixed with 3.0 mL ethanol and 0.3 mL DPPH• solution. The mixture was vortexed and incubated in the dark for 30 min at 25 ± 2 °C. Absorbance was measured at 517 nm using a UV–Vis spectrophotometer (SPECORD 200, Analytik Jena GmbH, Jena, Germany). The blank contained extract and ethanol without DPPH• to correct for background absorbance. The control contained DPPH• in ethanol without extract. Ascorbic acid (0.01–0.10 mg/mL) was used as the reference antioxidant to generate a standard calibration curve.

The percentage of radical scavenging was calculated as:

$$AA\%\;=\;100-\left[\frac{\left({Abs}_{sample}-\;{Abs}_{blank}\right)\times \;100}{{Abs}_{control}}\right]$$

where *A_control_* is the absorbance of the control, *A_sample_* is the absorbance of the reaction mixture, and *A_blank_* represents the absorbance of the extract blank. All measurements were performed in triplicate, and results are presented as mean ± standard deviation.

#### 2.7.2. Determination of IC_50_

The IC_50_ value, defined as the concentration of extract required to inhibit 50% of DPPH• radicals, was determined using a concentration–response assay. Serial dilutions of each extract (0.05–1.00 mg/mL) were prepared. In each reaction tube, 1 mL of diluted extract was mixed with 3 mL of 0.1 mM DPPH• solution in ethanol. After incubation in the dark for 30 min at 25 ± 2 °C, absorbance at 517 nm was recorded.

Radical-scavenging activity was calculated as:

$$\%\;inhibition=\left[\frac{A_{o}-A_{s}\;}{A_{o}}\right]\times 100$$

where *A_o_* is the absorbance of the control and *Aₛ* is the absorbance of the sample. The IC_50_ value was derived from the dose–response curve.

Dose–response curves were generated by plotting % inhibition against extract concentration. The IC_50_ value was obtained by nonlinear regression (four-parameter logistic model) using GraphPad Prism version 9.5.1 (GraphPad Software, San Diego, CA, USA). IC_50_ values for both extracts and ascorbic acid were expressed in mg/mL for consistency; the ascorbic acid IC_50_ was unit-corrected prior to conversion. All measurements were carried out in triplicate (technical) across three independent biological samples.

### 2.8. Statistics Analysis

All measurements were performed in four replicates, and results are presented as mean ± standard deviation (SD). Data from the different leaf-maturity stages were analyzed using one-way ANOVA to assess treatment effects. Prior to ANOVA, data were tested for normality using the Shapiro–Wilk test. Homogeneity of variance was assessed using Levene’s test to confirm that ANOVA assumptions were met. When significant differences were detected (*p* < 0.01), Fisher’s least significant difference (LSD) test was applied for pairwise comparisons. Statistical analyses were carried out using SPSS Statistics version 16.0 (IBM Corp., Armonk, NY, USA). Correlation analysis between total phenolic content (TPC) and IC_50_ values was performed using Pearson’s correlation coefficient to evaluate the relationship between phenolic concentration and antioxidant activity. This analytical approach ensured valid comparisons among leaf maturity stages and strengthened the interpretation of differences in phytochemical composition and antioxidant capacity.

## 3. Results and Discussion

### 3.1. Phytochemical Screening Analysis

The qualitative phytochemical composition of ethanolic extracts of *Tectona grandis* leaves at different maturity stages is presented in Table 1. The results reveal distinct variations in the presence, intensity, and distribution of secondary metabolites across leaf developmental stages. Secondary metabolites are low-molecular-weight organic compounds synthesized by plants. Although they are not essential for growth, they play key ecological and physiological roles. They exhibit multiple biological activities, including antioxidant, antimicrobial, and anti-inflammatory properties [23]. These compounds are generally classified as (A) terpenes, (B) phenolics, and (C) nitrogen- or sulfur-containing compounds. In the present study, ethanolic extracts of *T. grandis* leaves collected at four developmental stages (young, immature, mature, and mixed) were screened for their qualitative phytochemical composition. Results revealed clear variation among stages.

Tannins and coumarins were consistently detected in all samples. Tannins are potent radical-scavenging compounds that contribute significantly to antioxidant activity [24]. Coumarins, which are heterocyclic phytochemicals widely distributed in plants, display antimicrobial, anticoagulant, antioxidant, and anti-inflammatory effects [25]. Their presence in all extracts indicates that both tannins and coumarins are key contributors to the biological activities of teak leaves.

Stage-specific differences were also evident. Glycosides occurred in young and mature leaves, consistent with their recognized antibacterial and cardioprotective effects [26]. Alkaloids were present only in young leaves, suggesting developmental regulation. These nitrogenous compounds are associated with anti-inflammatory and antidiabetic actions. Saponins, detected exclusively in immature leaves, possess antimicrobial and insecticidal properties [27]. Flavonoids appeared predominantly in mixed-leaf extracts, aligning with their known roles as polyphenolic antioxidants with anti-inflammatory and anticancer activities [28]. In contrast, cardiac glycosides, terpenoids, anthraquinones, phlobatannins, and steroids were absent in all samples. This distribution pattern indicates that *T. grandis* leaves are dominated by polyphenolic and nitrogen-based metabolites rather than steroidal or terpenoid compounds.

Overall, the presence of tannins, coumarins, glycosides, alkaloids, saponins, and flavonoids demonstrates the chemical diversity of *T. grandis* leaves, with concentrations influenced by leaf maturity. Although this work focused on qualitative detection, the results provide biochemical support for the strong antioxidant and pharmacological properties of teak leaves. Future quantitative analyses using chromatographic techniques, such as HPLC or LC–MS, are warranted to identify and quantify key metabolites relevant to animal health applications.

### 3.2. Gas Chromatography–Mass Spectrometry (GC–MS) Analysis

The GC–MS analysis of ethanolic extracts from *T. grandis* leaves revealed clear differences in chemical profiles across the four developmental stages (Table 2, Table 3, Table 4 and Table 5). A wide range of compound classes was detected, including aldehydes, fatty acids, diterpenes, esters, and long-chain alcohols. Both the number and type of constituents varied according to leaf maturity. Only compounds that met the identification criteria specified in Section 2.4 were included as putative metabolites in Table 2, Table 3, Table 4 and Table 5. These criteria were: mass-spectral similarity (≥80% match), retention-index agreement, and consistent detection across replicates.

In addition to these metabolites, a small number of low-abundance peaks were detected in some chromatograms. These included trifluoroacetate esters, glycol-ether–related molecules, and other chemicals commonly reported as GC–MS laboratory artifacts, solvent impurities, or environmental contaminants rather than genuine plant constituents. Importantly, these same peaks were also present in the solvent blanks run under identical conditions. For transparency, they remain listed in Table 2, Table 3, Table 4 and Table 5 and are marked with a dagger (†). However, they were excluded from biological interpretation and do not influence the overall conclusions regarding the phytochemical composition of *T. grandis* leaves.

The GC–MS analysis demonstrated clear variation in the chemical profiles of young, medium, mature, and mixed *T. grandis* leaves. Young and medium leaves were characterized by aldehydes, fatty acids, and diterpenes that are widely associated with antioxidant, antimicrobial, and anti-inflammatory activities [29,30,31,32,33]. Phytol, a diterpene detected across all developmental stages, remained one of the dominant constituents and is well known for its antibacterial, antioxidant, anticancer, and immunostimulatory properties [33,39].

Mature leaves exhibited the greatest chemical diversity, reflecting more advanced secondary metabolism. Although some components, such as 3-methylglutaric anhydride and trifluoroacetic acid heptyl ester, remain poorly studied, several identified compounds—including 1-octyl trifluoroacetate, imidazolidinedione derivatives, and long-chain alcohols—have reported antimicrobial or anticonvulsant activities [31,35]. Additional oxygenated esters, such as ethanol 2-(dodecyloxy)- and methoxyacetic acid 2-tetradecyl ester, have also been linked to antimicrobial and anti-inflammatory effects [35].

Mixed leaves contained a diverse array of volatile esters and oxygenated molecules. Some volatiles lack documented pharmacological data. Others—including R(–)-3,7-dimethyl-1,6-octadiene, n-decanoic acid, and 2-propenoic acid 2-hydroxyethyl ester—exhibit antibacterial or antioxidant potential [37,41].

Collectively, these results highlight that leaf maturity influences the diversity and abundance of bioactive compounds. The recurrent detection of phytol, n-decanoic acid, and heptadecenal across all stages indicates their importance as core functional metabolites. Overall, the predominance of antioxidant and antimicrobial constituents aligns with the traditional uses of teak leaves. It also supports their potential application as a sustainable source of plant-derived bioactives for feed and nutraceutical industries.

### 3.3. Heavy Metal Analysis

The calibration parameters used for quantifying Pb, Cd, and As are presented in Table 6, and the concentrations measured in extracts from the four leaf-maturity stages are summarized in Table 7. Cadmium was detected at very low levels, and arsenic at trace concentrations, both of which were below the Thai Industrial Standards Institute (TISI) safety limit of 0.2 ppm [42,43].

Lead showed negative instrumental responses after baseline correction, indicating values below the detection limit. Such negative values are a well-documented methodological artifact in atomic absorption and plasma-based metal analyses. They typically result from baseline drifts, electronic noise, or slight matrix suppression effects rather than true metal concentrations. Because reagent blanks showed the same direction of drift, these readings were interpreted as being below the method detection limit and are therefore reported as ND (<LOD) in Table 7. This approach ensures that Pb is not misrepresented as having a measurable negative concentration.

Overall, the extremely low concentrations of Cd and As, together with non-detectable Pb, indicate minimal heavy metal contamination in the sampled leaves. However, these safety indications should be interpreted cautiously because Pb values fell below the detection limit and required correction to ND (<LOD). Therefore, the analytical limitations—particularly baseline drift and sensitivity constraints—prevent definitive conclusions about the absolute safety of the leaves. Toxic metals such as Pb and Cd are known to cause oxidative stress and bioaccumulate in animal tissues, while arsenic is recognized as a carcinogenic contaminant even at trace levels [44]. Although the present results demonstrate acceptably low heavy metal concentrations, future monitoring from different production environments would be beneficial to confirm consistency in safety for potential pharmacological or feed applications.

### 3.4. Analysis of Active Constituents

The quantitative composition of bioactive compounds in ethanolic extracts of *T. grandis* leaves at different maturity stages is presented in Table 8. Significant differences (*p* < 0.01) were observed in TPC, TFC, TTC, and CTC, indicating that leaf maturity strongly influences secondary-metabolite accumulation. TPC was lowest in young leaves and highest in mature leaves, with medium and mixed leaves showing intermediate values. A similar pattern was evident for TFC, which also peaked in mature leaves and declined in the mixed-leaf extracts (Table 8).

TTC values also varied significantly (*p* < 0.01). The highest TTC was observed in medium leaves, followed by mixed, young, and mature leaves (Table 8). In contrast, CTC showed a different pattern, with mature leaves exhibiting the greatest levels, young and medium leaves at intermediate levels, and mixed leaves the lowest. The enhanced accumulation of phenolics and flavonoids in mature leaves likely results from upregulation of the phenylpropanoid pathway. In this pathway, phenylalanine ammonia-lyase (PAL) converts phenylalanine into cinnamic acid, which serves as a precursor for multiple phenolic compounds [45]. Phenolics protect plant cells by donating hydrogen atoms or electrons to neutralize reactive oxygen species (ROS). They also chelate transition metals (Fe^2+^, Cu^2+^), thereby preventing radical formation [46,47]. Flavonoids complement these antioxidant effects by modulating redox signaling, enhancing activities of antioxidant enzymes such as superoxide dismutase and catalase, and inhibiting lipid peroxidation. Chávez-Salgado et al. [48] and Pepi Budianto et al. [49] reported similar antioxidant and antifungal activities and comparable TPC and TFC values in ethanolic teak extracts. Tannins, another major class of polyphenols, also showed maturity-dependent variation. Their ability to bind proteins and metal ions enhances their radical-scavenging properties and contributes to the inhibition of oxidative enzymes [50]. CTC, which represent polymeric flavan-3-ols, were again most abundant in mature leaves, consistent with reports in *Terminalia nigrovenulosa* and *Halimium halimifolium* using the Broadhurst et al. method [51]. From a mechanistic standpoint, the coordinated increase in TPC, TFC, and CTC in mature leaves is consistent with activation of the phenylpropanoid pathway under prolonged exposure to light and oxidative cues. In this pathway, phenylalanine ammonia-lyase (PAL) converts phenylalanine into cinnamic acid, which serves as a precursor for a wide range of phenolic acids, flavonoids, and tannin monomers [45]. These metabolites act as chain-breaking antioxidants by donating hydrogen atoms or single electrons to terminate radical reactions. Their multiple hydroxyl groups also enable chelation of transition metals such as Fe^2+^ and Cu^2+^, thereby limiting Fenton-type chemistry and hydroxyl-radical formation [46,47]. In biological systems, tannin–protein and tannin–metal complexes can further modulate oxidative processes by protecting susceptible proteins from oxidative modification and altering the activity of pro-oxidant enzymes [48]. Together, these biochemical interactions explain why leaves with higher phenolic and tannin contents exhibit stronger antioxidant behavior. Overall, these findings demonstrate that *T. grandis* leaves accumulate increasing levels of phenolics, flavonoids, and tannins as part of a coordinated antioxidant defense system. These metabolites function synergistically to donate electrons or hydrogen atoms, chelate pro-oxidant metals, and stabilize cellular membranes, collectively mitigating oxidative stress and inflammation. Their maturity-related increase highlights *T. grandis* as a promising and renewable source of natural antioxidants suitable for nutraceutical and functional-feed applications.

### 3.5. Antioxidant Screening Analysis

Reactive oxygen species (ROS) and reactive nitrogen species (RNS) are normal metabolic by-products that, when produced in excess, cause oxidative stress and cellular damage [52]. Plant-derived antioxidants play a central role in neutralizing these radicals and restoring redox balance [46]. The ethanolic extracts of *T. grandis* leaves at different maturity stages were therefore evaluated for antioxidant capacity using inhibition-of-oxidant and DPPH radical-scavenging assays. Ascorbic acid was used as the standard reference.

As shown in Table 9, mature-leaf extracts exhibited the highest oxidant-inhibition activity and slightly exceeded the activity of ascorbic acid. Young, mixed, and medium leaves showed significantly lower inhibition (*p* < 0.01). The superior performance of mature leaves corresponds with their elevated phenolic and flavonoid contents (Table 8). Phenolics function through hydrogen-atom transfer (HAT) and single-electron transfer (SET) mechanisms, neutralizing free radicals and stabilizing oxidative intermediates. They also chelate metals such as Fe^2+^ and Cu^2+^ to prevent hydroxyl-radical formation. Flavonoids enhance protection via activation of Nrf2–ARE-regulated antioxidant enzymes and suppression of pro-oxidant enzymes [46].

These results agree with Naira Nayeem [53], who observed similar antioxidant activity (94.51%) in methanolic and aqueous *T. grandis* extracts. The present study confirms that mature and young leaves are strong natural antioxidant sources suitable for further bioactive development [54].

The corrected IC_50_ value for ascorbic acid (0.0767 mg/mL) confirms the expected high antioxidant potency of the pure standard compared with the crude ethanolic extracts of *T. grandis* leaves (Table 10). Among the extracts, mature leaves demonstrated the strongest DPPH radical-scavenging activity, as indicated by the lowest IC_50_, whereas young leaves showed the weakest activity. At a mechanistic level, this strong inverse relationship between TPC and IC_50_ suggests that phenolic-rich extracts are more efficient at interrupting radical chain reactions. Phenolic hydroxyl groups can donate hydrogen atoms to DPPH• and related radicals, forming resonance-stabilized phenoxyl intermediates that do not propagate further oxidative damage [46]. In parallel, flavonoid and condensed-tannin structures are known to interact with oxidoreductive enzymes and membrane lipids, dampening lipid peroxidation and supporting the activity of endogenous antioxidant systems such as superoxide dismutase, catalase, and glutathione peroxidase [47,50]. The higher phenolic load in mature leaves therefore provides a plausible biochemical basis for their superior radical-quenching performance in vitro. To verify this relationship statistically, a correlation analysis was performed using the expanded dataset that included all biological replicates. The results showed a strong and statistically significant negative correlation between TPC and IC_50_ values (r = −0.85, *p* < 0.01). This indicates that extracts with higher phenolic concentrations consistently exhibited stronger radical-scavenging activity and confirms that phenolics are major contributors to the antioxidant behavior of *T. grandis* leaf extracts.

The corrected values restore the anticipated relationship between standards and plant extracts: ascorbic acid shows substantially greater potency, while the crude extracts exhibit moderate activity typical of phytochemical mixtures. The superior performance of the mature-leaf extract reflects its enriched concentrations of hydroxylated phenolics and condensed tannins, which effectively donate electrons or hydrogen atoms to stabilize DPPH radicals. Given these antioxidant properties, the extract may serve as a promising natural additive for livestock feed [55]. It may help improve oxidative stability of diets and support animal health under production stressors.

## 4. Conclusions

This study demonstrated that *T. grandis* leaves contain a diverse array of phytochemicals and show measurable antioxidant activity across all maturity stages. Heavy metal concentrations were below detection limits under the analytical conditions used. However, safety conclusions remain preliminary due to limitations in Pb detectability and instrumental sensitivity. Mature leaves showed the highest accumulation of phenolics, flavonoids, and tannins, which corresponded with their strongest DPPH radical-scavenging activity and oxidant-inhibition capacity. Compounds such as phytol and n-decanoic acid were consistently detected across developmental stages, suggesting their relevance as core contributors to the antioxidant profile of teak leaves.

While the findings provide a useful baseline for understanding the chemical composition and antioxidant potential of *T. grandis* leaves, this work is limited to chromatographic profiling and in vitro antioxidant assays. Consequently, any implications for biological efficacy, safety, or feed application should be interpreted with caution. Moreover, several low-abundance peaks detected by GC–MS were identified as laboratory artifacts; future studies incorporating HPLC/LC–MS quantification and rigorous blank-control validation are recommended to strengthen compound identification.

To advance the practical relevance of this research, additional investigations are required. These include dose–response assays, targeted phenolic quantification, in vitro rumen-fermentation studies, and in vivo feeding trials. Collectively, these steps will help clarify the biological significance of the identified compounds and determine the suitability of *T. grandis* leaf extracts for nutraceutical or livestock-feed applications.

## Figures and Tables

**Table 1:** Qualitative phytochemical composition of ethanolic extracts of *Tectona grandis* leaves at different maturity stages.

Class of Compounds	Leaves Ethanolic Extract			
	Young Leaves	Immature Leaves	Mature Leaves	Mixed Leaves
Cardiac glycoside	−	−	−	−
Flavonoids	−	−	−	+
Saponins	−	+	−	−
Tannins	+	+	+	+
Terpenoids	−	−	−	−
Antraquinones	−	−	−	−
Coumarin	+	+	+	+
Phlobatannins	−	−	−	−
Steroids	−	−	−	−
Alkaloids	+	−	−	−
Glycoside	+	−	+	−

**Note:** (+) Present, (−) Absent.

**Table 2:** GC–MS profile of bioactive compounds identified in ethanolic extract of young *Tectona grandis* leaves.

No.	RT	Compound	Formula	Mw	Peak Area%	Pharmacological Action	Reference
1	14.46	Butanoic acid, 1,1-dimethylethyl ester	C_8_H_16_O_2_	144	0.48	No report available	-
2	28.68	14-Heptadecenal	C_17_H_32_O	252	4.62	Bioactive phytochemicals, a source of bioactive compounds in pharmaceutical industries, antioxidant activity, antimicrobial, and anti-inflammatory activity, antifungal	[29]
3	33.30	n-Decanoic acid	C_10_H_20_O_2_	172	4.93	Antibacterial and antifungal	[30]
4	44.70	Thunbergol	C_20_H_34_O	290	44.50	Diterpene, antimicrobial, Larvicidal properties	[31]

All compounds were identified based on their mass-spectral similarity with the NIST17 library (minimum similarity index ≥ 80%). Retention indices (RIs) were calculated using a homologous n-alkane series under the same chromatographic conditions and compared with published reference RI values for Rtx-5MS columns. Only compounds showing acceptable agreement between experimental and reported RI values were included. Identifications follow accepted GC–MS metabolite-annotation standards and are considered putative but reliable.

**Table 3:** GC–MS profile of bioactive compounds identified in ethanolic extract of medium *Tectona grandis* leaves.

No.	RT	Compound	Formula	Mw	Peak Area%	Pharmacological Action	Reference
1	28.69	16-Heptadecenal	C_17_H_32_O	252	6.38	Bioactive compounds, antioxidant, anti-inflammatory, antimicrobial, antidiabetic, hepatoprotective anticancer	[32]
2	33.31	n-Hexadecanoic acid	C_16_H_32_O_2_	256	14.05	Bioactive compounds, antioxidant, anti-inflammatory, antimicrobial, antidiabetic, hepatoprotective anticancer	[32,33]
3	38.08	Phytol	C_20_H_40_O	296	7.81	Bioactive compounds, antibacterial and antioxidant activity	[33]

All compounds were identified based on their mass-spectral similarity with the NIST17 library (minimum similarity index ≥ 80%). Retention indices (RIs) were calculated using a homologous n-alkane series under the same chromatographic conditions and compared with published reference RI values for Rtx-5MS columns. Only compounds showing acceptable agreement between experimental and reported RI values were included. Identifications follow accepted GC–MS metabolite-annotation standards and are considered putative but reliable.

**Table 4:** GC–MS profile of bioactive compounds identified in ethanolic extract of mature *Tectona grandis* leaves.

No.	RT	Compound	Formula	Mw	Peak Area%	Pharmacological Action	Reference
1	9.13	1-Octyl trifluoroacetate	C_10_H_17_F_3_O_2_	226	4.82	Esters, antimicrobial	[31]
2	9.42	3-Methylglutaric anhydride	C_6_H_8_O_3_	128	0.59	No report available	-
3	9.48	Carbonic acid, propargyl 2-ethylhexyl ester	C_12_H_20_O_3_	212	1.53	No report available	-
4	9.71	Trifluoroacetic acid, heptyl ester	C_9_H_15_F_3_O_2_	212	1.52	No report available	-
5	11.89	2,4-Imidazolidinedione, 5,5-dimethyl-	C_5_H_8_N_2_O_2_	128	2.38	Anticonvulsant and antiepileptic	[34]
6	14.27	1-Heptanol	C_7_H_16_O	116	1.52	Long-chain fatty alcohol, antibacterial	[35]
7	16.33	1-Dodecanol	C_12_H_26_O	186	28.56	Long-chain fatty alcohol, volatile compounds, antibacterial	[35]
8	16.97	Nitric acid, nonyl ester	C_9_H_19_NO_3_	189	6.29	Bioactive natural compounds and chemical constituents	[36]
9	22.05	1-Heptanol, 6-methyl-	C_8_H_18_O	130	4.91	No report available	-
10	23.69	Ethanol, 2-(dodecyloxy)-	C_14_H_30_O_2_	230	17.12	Volatile compounds, anti-microbial activity, anti-inflammatory	[37]
11	31.70	Octane, 2-bromo-	C_8_H_17_Br	192	3.37	No report available	-
12	34.22	Methoxyacetic acid, 2-tetradecyl ester	C_17_H_34_O_3_	286	5.59	Volatile compounds, antibacterial	[38]
13	38.10	Phytol	C_20_H_40_O	296	5.09	Diterpene, antibacterial, anticancer, anti-inflammatory, anti-diuretic, immunostimulatory and anti-diabetic	[31,39]
14	40.66	Ethylene glycol monoisobutyl ether	C_6_H_14_O_2_	118	0.82	No report available	-
15	42.71	2-Butenoic acid, 2-methoxy-, methyl ester, (Z)-	C_6_H_10_O_3_	130	0.84	No report available	-

All compounds were identified based on their mass-spectral similarity with the NIST17 library (minimum similarity index ≥ 80%). Retention indices (RIs) were calculated using a homologous n-alkane series under the same chromatographic conditions and compared with published reference RI values for Rtx-5MS columns. Only compounds showing acceptable agreement between experimental and reported RI values were included. Identifications follow accepted GC–MS metabolite-annotation standards and are considered putative but reliable.

**Table 5:** GC–MS profile of bioactive compounds identified in ethanolic extract of mixed *Tectona grandis* leaves.

No.	RT	Compound	Formula	Mw	Peak Area%	Pharmacological Action	Reference
1	9.32	Oxalic acid, butyl propyl ester	C_9_H_16_O_4_	188	0.54	No report available	-
2	14.47	2,2,4-Trimethyl-3-pentanone	C_8_H_16_O	128	0.72	No report available	-
3	28.69	R(-)3,7-Dimethyl-1,6-octadiene	C_10_H_18_	138	9.22	Antibacterial activity	[40]
4	33.31	n-Decanoic acid	C_10_H_20_O_2_	172	25.05	Antioxidant potential, anti-inflammatory, antibacterial, antifungal activity	[41]
5	33.42	2-Propenoic acid, 2-hydroxyethyl ester	C_5_H_8_O_3_	116	1.30	Volatile organic compounds, antioxidant	[35]
6	39.80	4-Trifluoroacetoxyoctane	C_10_H_17_F_3_O_2_	226	0.79	Medicinal plants, antibacterial activity, antifungal activity	[42]

All compounds were identified based on their mass-spectral similarity with the NIST17 library (minimum similarity index ≥ 80%). Retention indices (RIs) were calculated using a homologous n-alkane series under the same chromatographic conditions and compared with published reference RI values for Rtx-5MS columns. Only compounds showing acceptable agreement between experimental and reported RI values were included. Identifications follow accepted GC–MS metabolite-annotation standards and are considered putative but reliable.

**Table 6:** Calibration curves and analytical parameters for heavy metal determination (Pb, Cd, As) in *Tectona grandis* leaves using atomic absorption spectrophotometry (AAS).

Sample	Cd (ppm)	Pb (ppm)	As (ppb)
Equation	y = 0.7593x + 7 × 10^−6^	y = 0.0215x + 0.0042	y = 0.0086x + 0.0051
R^2^	0.997	0.999	0.999
SD	0.156	0.176	0.128
LOD	0.005	0.003	−0.657
LOQ	0.013	0.031	2.914

R^2^ = coefficient of determination, indicating the goodness of fit of the calibration curve; SD = standard deviation of replicate measurements; LOD = limit of detection, calculated as 3.3 × (SD/slope); LOQ = limit of quantification, calculated as 10 × (SD/slope).

**Table 7:** Concentrations of cadmium (Cd), lead (Pb), and arsenic (As) in ethanolic extracts of *Tectona grandis* leaves at different maturity stages (mean ± SD). Values below the limit of detection (LOD) are reported as <LOD.

Sample	*Tectona grandis* Linn.											
	Young Blade			Medium Blade			Mature Blade			Mixed Blade		
	Conc.	Sd	%RSD	Conc.	Sd	%RSD	Conc.	Sd	%RSD	Conc.	Sd	%RSD
Cd (ppm)	0.004	0.000	11.500	0.001	0.000	7.500	0.001	0.000	8.650	0.003	0.000	3.536
Pb (ppm)	<LOD	0.009	4.094	<LOD	0.003	1.135	<LOD	0.011	5.215	<LOD	0.008	3.431
As (ppb)	17.593	0.016	0.124	16.593	0.018	0.141	12.488	0.028	0.217	14.861	0.032	0.251

LOD and LOQ values for the AAS method were as follows: Cd = LOD 0.001 mg/kg, LOQ 0.003 mg/kg; Pb = LOD 0.005 mg/kg, LOQ 0.015 mg/kg; As = LOD 0.010 mg/kg, LOQ 0.030 mg/kg. Values below the limit of detection (LOD) are reported as <LOD. Cd and Pb are expressed in ppm (mg/kg), and As is expressed in ppb according to the instrument calibration range.

**Table 8:** Total phenolic, flavonoid, tannin, and condensed tannin contents in ethanolic extracts of *Tectona grandis* leaves at different maturity stages.

Sample	Total Phenolic Content (mg GAE/g DW)	Total Flavonoid Content (mg QE/g DW)	Total Condensed Tannins (mg CE/g DW)	Total Tannins (mg GAE/g Extract)
Young leaves	5.183 ± 0.0024 ^d^	0.082 ± 0.00017 ^d^	0.286 ± 0.00042 ^b^	0.316 ± 0.00033 ^c^
Medium leaves	6.595 ± 0.0041 ^c^	0.208 ± 0.00023 ^b^	0.237 ± 0.00037 ^c^	0.467 ± 0.00028 ^a^
Mature leaves	8.751 ± 0.0187 ^a^	0.359 ± 0.0173 ^a^	0.303 ± 0.00041 ^a^	0.310 ± 0.00047 ^d^
Mixed leaves	6.986 ± 0.0053 ^b^	0.136 ± 0.00026 ^c^	0.088 ± 0.00039 ^d^	0.366 ± 0.00044 ^b^
*p*-value	<0.001	<0.001	<0.001	<0.001

Letters indicating statistical differences (^a^, ^b^, ^c^, ^d^) are included, but the table footnotes are unclear. It is necessary to ensure consistency between letters and ANOVA outcomes.

**Table 9:** Inhibition of oxidant activity (%) of ethanolic extracts of *Tectona grandis* leaves at different maturity stages compared with ascorbic acid.

Sample	% Antioxidant
Ascorbic acid	94.991 ± 1.470 ^a^
Young leaves	89.050 ± 0.649 ^b^
Medium leaves	86.627 ± 0.062 ^c^
Mature leaves	95.877 ± 0.123 ^a^
Mixed leaves	88.534 ± 0.035 ^b^
*p*-value	<0.0000

Letters indicating statistical differences (^a^, ^b^, ^c^) are included, but the table footnotes are unclear. It is necessary to ensure consistency between letters and ANOVA outcomes.

**Table 10:** DPPH radical-scavenging capacity (IC_50_, mg/mL) of ethanolic extracts of *Tectona grandis* leaves at different maturity stages compared with ascorbic acid.

Sample	IC_50_ (mg/mL)
Ascorbic acid	0.0767 ± 0.0026
Young leaves	50.348 ± 0.167
Medium leaves	44.359 ± 0.192
Mature leaves	22.928 ± 0.111 ^a^
Mixed leaves	24.235 ± 0.291 ^ab^
*p*-value	0.001

Letters indicating statistical differences (^a^, ^b^) are included, but the table footnotes are unclear. It is necessary to ensure consistency between letters and ANOVA outcomes.

## Data Availability

The data supporting the findings of this study are available from the corresponding author upon reasonable request.
